# Diagnosis and Surveillance of West Nile Virus Infection in Horses: Current Methods, Challenges, and Future Directions

**DOI:** 10.3390/vetsci13040332

**Published:** 2026-03-30

**Authors:** Paula Nistor, Livia Stanga, Vlad Iorgoni, Alexandru Gligor, Alexandru Ciresan, Horia Iorgoni, Bogdan Florea, Vlad Cocioba, Ionica Iancu, Cosmin Horatiu Maris, Beata Nowicka, Viorel Herman

**Affiliations:** 1 Department of Infectious Diseases and Preventive Medicine, Faculty of Veterinary Medicine, University of Life Sciences “King Mihai I” from Timişoara, 300645 Timişoara, Romania; paula.nistor@usvt.ro (P.N.); vlad.iorgoni@usvt.ro (V.I.); alexandru.gligor@usvt.ro (A.G.); ionica.iancu@usvt.ro (I.I.); viorel.herman@fmvt.ro (V.H.); 2 Doctoral School “Veterinary Medicine”, University of Life Sciences “King Mihai I” from Timişoara, Calea Aradului 119, 300645 Timişoara, Romania; bogdan-alexandru.florea.fmv@usvt.ro (B.F.); vlad-mihai.cocioba.fmv@usvt.ro (V.C.); 3 Discipline of Microbiology, Faculty of Medicine, “Victor Babes” University of Medicine and Pharmacy, Eftimie Murgu Square 2, 300041 Timişoara, Romania; 4 Department of Surgery, Faculty of Veterinary Medicine, University of Life Sciences “King Mihai I” from Timişoara, 300645 Timişoara, Romania; alexandru.ciresan@usvt.ro; 5 Faculty of Physical Education and Sport, West University of Timișoara, 300223 Timișoara, Romania; horia.iorgoni@e-uvt.ro; 6 Department of Internal Medicine, University of Life Sciences “King Mihai I” from Timişoara, 300645 Timişoara, Romania; 7 Department of Animal Husbandry, University of Life Sciences “King Mihai I” from Timişoara, 300645 Timişoara, Romania; 8 Department of Forestry, Faculty of Engineering and Applied Technologies, University of Life Sciences “King Mihai I” from Timișoara, 300645 Timișoara, Romania; cosmin.maris@usvt.ro; 9 Department and Clinic of Animal Surgery, Faculty of Veterinary Medicine, University of Life Sciences in Lublin, 20-612 Lublin, Poland; beatanowicka@aol.com; 10 Academy of Romanian Scientists (AOSR), Splaiul Independenței 54, 050094 Bucharest, Romania

**Keywords:** West Nile virus, horses, diagnosis, serology, surveillance, One Health, Europe, sentinel animals

## Abstract

West Nile virus (WNV) is a mosquito-borne flavivirus of growing importance for both human and equine health in Europe. Horses are highly susceptible to neurological disease and, because they share ecological exposure with humans, they represent valuable sentinels for detecting local viral circulation within a One Health framework. However, diagnosis of WNV infection in equines is complicated by the short and low-level viraemia, which limits the sensitivity of molecular assays, and by serological cross-reactivity with related flaviviruses and the confounding effects of vaccination. In this narrative review, we summarise the current diagnostic tools for WNV in horses, including direct detection methods (RT-qPCR, virus isolation, antigen detection) and indirect serological approaches (IgM and IgG ELISA, virus neutralisation tests), and discuss their practical performance and constraints in clinical and surveillance settings. We further examine equine surveillance systems, passive clinical reporting, active serosurveys and sentinel cohorts, and their integration with vector, avian and environmental monitoring. Key challenges include methodological heterogeneity, limited access to confirmatory testing and variable cross-sector data sharing. Finally, we outline future directions, highlighting the need for harmonised laboratory protocols, innovative field-deployable diagnostics, genomic surveillance and integrated, multi-source monitoring systems to strengthen early warning capacity and improve preparedness for WNV outbreaks in equine populations.

## Simple Summary

West Nile virus (WNV) is a mosquito-borne pathogen that affects both humans and horses, with the latter being particularly susceptible to neurological disease. Because horses share environmental exposure with humans, they can serve as early indicators of local virus circulation within a One Health framework. Diagnosis of WNV infection in horses is challenging due to the short and low-level viraemia, which limits the sensitivity of molecular tests. As a result, diagnosis relies mainly on serological methods, although these are affected by cross-reactivity with related flaviviruses and vaccination. This review summarises current diagnostic approaches and surveillance strategies in horses, highlighting their practical limitations. It also outlines key challenges and future priorities, including the need for improved diagnostic tools, standardised protocols and better integration of animal, vector and environmental data to strengthen early detection and outbreak preparedness.

## 1. Introduction

West Nile virus (WNV) is an arthropod-borne zoonosis of increasing relevance for both human and veterinary public health [1]. The virus, a member of the family *Flaviviridae* and genus *Orthoflavivirus*, circulates primarily in an enzootic cycle involving avian reservoir hosts and *Culex* mosquitoes [2]. The spillover to incidental hosts such as humans and equines occurs when viral amplification increases, although these hosts typically develop viraemia insufficient for sustaining onward transmission [3].

Equines are particularly vulnerable to WNV and may develop severe neurological disease, with case fatality rates surpassing 25–30% in some outbreaks. Because horses share ecological niches with humans and respond immunologically in a measurable way, they are regarded as valuable sentinel species [4]. The detection of seroconversion or clinical disease in equines often mirrors ongoing regional viral circulation and supports early warning frameworks within a One Health context [5].

Over the past decade, the epidemiology of WNV in Europe has shifted markedly. Lineage 2 has become widely established across Central and Eastern Europe, while climate change, migratory bird dynamics, vector adaptation and environmental modifications have contributed to an increased frequency, intensity and geographic expansion of outbreaks [6]. Recent European reports document widespread human and equine cases and highlight a growing need for harmonised monitoring [7].

Despite their epidemiological importance, equine surveillance systems remain fragmented across Europe. Variations in diagnostic capacity, laboratory methodology, reporting systems and sampling approaches result in inconsistent datasets and reduced comparability between countries [8,9]. Diagnostic challenges further complicate surveillance, e.g., equine viraemia is typically low and short-lived, limiting RT-QPCR sensitivity, whereas serology is influenced by cross-reactivity with related flaviviruses, prior exposure and vaccination [10,11,12].

In this context, a critical synthesis of current diagnostic tools, surveillance strategies and operational challenges is essential. This review aims to (i) evaluate available diagnostic methods for WNV infection in equines; (ii) summarise existing surveillance approaches; (iii) discuss technical and epidemiological constraints affecting diagnostic performance and data interpretation; and (iv) identify future priorities for improving WNV monitoring within a One Health framework.

## 2. Epidemiology and Biological Background

WNV is a mosquito-borne flavivirus belonging to the family *Flaviviridae*, genus *Orthoflavivirus*, and a member of the Japanese encephalitis serocomplex. The virus circulates primarily in an enzootic cycle between avian reservoir hosts, especially migratory and resident passerines, and ornithophilic mosquitoes, predominantly *Culex* spp. [13,14]. Birds develop high viraemia that supports viral amplification, while incidental hosts such as equines and humans usually develop insufficient viraemia to contribute to transmission, classifying them as “dead-end” hosts [15].

### 2.1. Viral Lineages and Genetic Evolution

Several genetic lineages of WNV have been described, but lineages 1 and 2 are the most epidemiologically relevant in Europe. Historically, lineage 1 was associated with major outbreaks reported in the early 2000s, whereas lineage 2 has become predominant in many parts of Central and Eastern Europe in recent years [6,16,17]. In southern Europe, the co-circulation of lineages 1 and 2 has also been documented, indicating continued viral movement along migratory bird routes and ongoing local evolution [18,19].

### 2.2. Transmission Dynamics and Ecological Drivers

Transmission intensity is shaped by interactions between vectors, avian reservoirs and environmental conditions [20]. In Europe, *Culex pipiens*, *Culex modestus* and *Culex perexiguus* are considered the principal vectors involved in enzootic and spillover transmission [20,21,22,23]. Environmental determinants, including temperature, precipitation, humidity, presence of wetlands, irrigated agriculture and urban water sources, directly influence mosquito abundance and feeding behaviour [24]. Warmer summers and mild autumns reduce the extrinsic incubation period of the virus and extend the seasonal transmission window, thereby amplifying outbreak risk [25,26].

**Figure 1:**
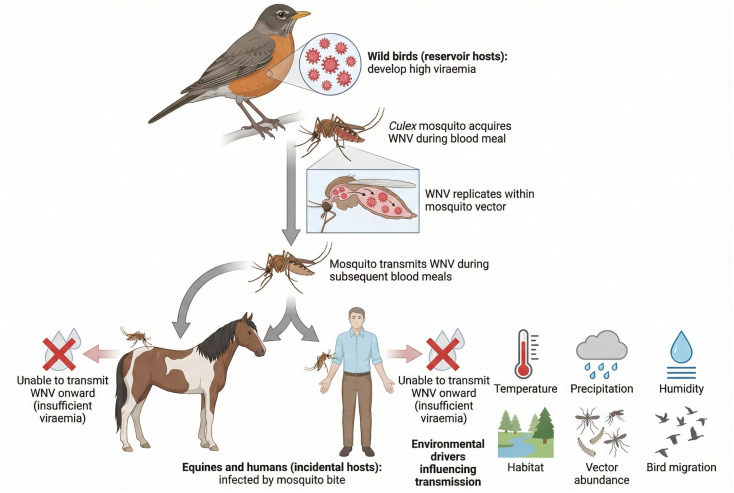
The transmission cycle of WNV involving avian hosts, mosquito vectors and incidental equine and human hosts. This schematic illustrates the classical transmission cycle of WNV. Birds serve as amplifying hosts, maintaining high levels of viraemia capable of infecting mosquito vectors, primarily *Culex* spp. Infected mosquitoes subsequently transmit the virus during blood feeding. Equines and humans become infected as incidental dead-end hosts, as their viraemia is insufficient to support further transmission. Environmental factors such as temperature, precipitation and habitat characteristics influence vector abundance and viral amplification.

Migratory birds play a critical role in the long-distance movement of WNV across regions [19]. Europe lies along major flyways that connect Africa, the Middle East and Eurasia, allowing repeated viral introductions, lineage mixing and geographic expansion of both established and emerging strains [27,28,29,30]. The classical WNV transmission cycle involving avian hosts, mosquito vectors and incidental equine and human hosts is summarised in Figure 1.

### 2.3. Equines as Sentinel and Incidental Hosts

Although most equine infections remain subclinical, clinically affected horses may develop severe neurological disease, including ataxia, cranial nerve deficits, paresis, recumbency and, in some outbreaks, mortality rates of 23–43% [31]. Because horses share environments with humans and are highly exposed to mosquito vectors, equine infections frequently coincide with or precede human cases. These characteristics underpin their use as sentinel species for detecting viral circulation within a One Health surveillance context [32].

Seroprevalence studies in Europe show substantial geographic variation. Investigations in high-risk regions of Central and Eastern Europe have documented equine seroprevalence ranging between approximately 4% and 15% in unvaccinated populations, with higher values in wetland- and river-associated ecosystems [11,31]. Such findings underscore the importance of ecological context when interpreting equine serological data.

### 2.4. Current European Situation (2021–2025)

During the past decade, Europe has experienced increased frequency, intensity and geographic spread of WNV activity. Several countries in southern, central and eastern Europe report annual outbreaks in humans, horses and birds, with occasional incursions into previously unaffected regions [11,31,33]. Climatic trends, vector adaptation to peri-urban environments, ecological changes and variable surveillance capacity all contribute to the heterogeneous distribution of cases across the continent [20,34,35].

In addition, the circulation of related flaviviruses, particularly Usutu virus, complicates clinical and serological interpretation, further highlighting the need for harmonised diagnostic strategies and integrated surveillance systems [33,36].

## 3. Diagnostic Methods in Equines

Diagnostic evaluation of WNV infection in horses relies on a combination of direct and indirect methods, each presenting specific strengths and limitations when applied to equine cases [37]. Because viraemia in horses is typically short-lived and of low magnitude, direct viral detection is often challenging [38]. Consequently, serological assays constitute the backbone of equine WNV diagnosis, although their interpretation requires careful consideration of timing, vaccination status and potential cross-reactivity with other flaviviruses [39,40].

### 3.1. Direct Detection Methods

#### 3.1.1. Molecular Assays (RT-qPCR/Real-Time RT-qPCR)

Reverse transcription polymerase chain reaction (RT-qPCR) is the preferred method for detecting WNV RNA in whole blood, serum, cerebrospinal fluid (CSF) or post-mortem central nervous system (CNS) tissues [41]. Real-time RT-qPCR platforms provide improved sensitivity, specificity and the ability to differentiate between WNV lineages [42].

However, in equines, viraemia is transient and often falls below the limit of detection at the time neurological signs appear [43]. As a result, negative RT-qPCR results do not reliably exclude infection [44,45]. External quality assessment studies have shown strong performance for lineage-specific assays, whereas broad-range flavivirus RT-qPCRs tend to be less sensitive in low-titre samples [46,47,48].

#### 3.1.2. Virus Isolation and Antigen Detection

Virus isolation in cell culture and antigen detection via immunohistochemistry (IHC) are definitive diagnostic tools but are infrequently used in routine equine diagnostics. Virus isolation requires biosafety level 3 (BSL-3) facilities, significant time and high-quality samples, making it impractical for clinical decision making [9]. IHC performed on CNS tissues from fatal cases can provide confirmation even when viral RNA is degraded, but its use is necessarily limited to post-mortem scenarios [49].

### 3.2. Indirect Detection Methods (Serology)

Because direct detection frequently fails in clinical equine cases, serology is essential for identifying recent or past exposure to WNV [8].

#### 3.2.1. IgM Capture ELISA

The detection of WNV-specific IgM antibodies in serum or CSF remains the most reliable indicator of recent infection. IgM antibodies appear shortly after infection and typically persist for several weeks. IgM ELISAs demonstrate good specificity but variable sensitivity, particularly in samples with low antibody titres or when collection is delayed. Despite these limitations, IgM ELISA is the primary diagnostic tool for clinically suspected acute cases. Most IgM ELISA assays are based on the detection of antibodies directed against the viral envelope (E) protein, which represents the main immunodominant antigen in flavivirus infections [50,51].

#### 3.2.2. IgG ELISA and Competitive ELISA Platforms

IgG-based assays, including competitive or epitope-blocking ELISAs, are widely used in equine serosurveys and for assessing cumulative exposure. These tests are highly sensitive and suitable for large-scale screening [52]. However, IgG assays cannot distinguish between recent and past infection, nor can they differentiate natural infection from vaccine-induced immunity [53]. Their interpretation is further complicated by cross-reactivity with antigenically related flaviviruses such as Usutu virus, which co-circulates in several European regions [52,53]. IgG-based assays, including competitive or epitope-blocking ELISAs, are widely used in equine serosurveys and for assessing cumulative exposure. These assays typically target epitopes of the viral envelope (E) protein, although some newer platforms incorporate NS1-based antigens to improve specificity and reduce cross-reactivity [40,41,42,43,44,45,46,47,48,49,50,51,52,53].

#### 3.2.3. Virus Neutralisation Test (VNT/PRNT)

Neutralisation assays remain the gold standard for confirming WNV-specific antibodies due to their high specificity and ability to resolve serological cross-reactivity. VNT/PRNT assays are particularly important for validating IgG ELISA findings in areas where multiple flaviviruses are present [54]. Their limitations include labour intensity, the requirement for high-containment facilities, longer turnaround times and restricted availability in routine diagnostic laboratories.

### 3.3. Interpretation of Diagnostic Results

Accurate interpretation requires integrating laboratory findings with clinical presentation, epidemiological context and sampling timing. A positive IgM ELISA or detectable viral RNA strongly supports recent infection [55]. Conversely, the presence of IgG alone, especially in a clinically healthy horse, typically reflects historical exposure or vaccination rather than active disease. In vaccinated populations, serological interpretation must be approached cautiously due to the inability of many assays to differentiate immune responses [56].

### 3.4. Proposed Diagnostic Workflow

**Figure 2:**
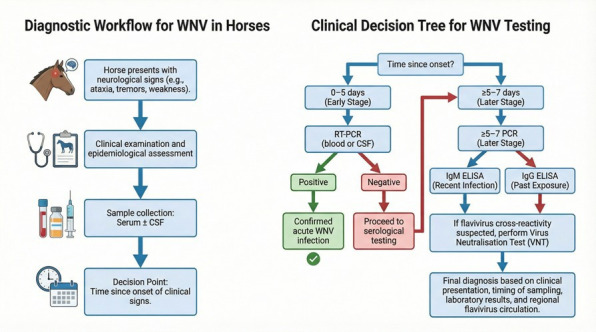
Proposed diagnostic workflow for WNV infection in equines. This flow diagram outlines a stepwise diagnostic approach for suspected WNV infection in horses. Following the identification of compatible neurological signs, serum and CSF should be collected. Early-stage infection warrants RT-qPCR testing, whereas IgM ELISA serves as the primary indicator of recent infection. IgG ELISA supports assessment of past exposure, and virus neutralisation testing provides confirmatory specificity where cross-reactivity is suspected. Diagnostic interpretation must integrate clinical context, timing of sampling and regional flavivirus circulation.

A practical diagnostic approach begins with early sampling of serum and, when feasible, CSF in horses presenting with compatible neurological signs [57]. During early infection, simultaneous RT-QPCR and IgM ELISA testing is recommended. Positive IgM or PCR results indicate probable acute WNV infection [58]. When both tests are negative but clinical suspicion remains, IgG ELISA followed by VNT confirmation can clarify exposure history [57]. For seroepidemiological studies, IgG ELISA is the preferred screening tool, with a subset of positive samples subjected to VNT to ensure specificity [50]. Based on these considerations, a practical diagnostic workflow is proposed (Table 1; Figure 2).

**Table 1:** Diagnostic methods for WNV infection in equines: principles, sample types, advantages and limitations.

Diagnostic Method	Principle/Target	Sample Types	Advantages	Limitations (Especially in Equines)
Real-time RT-qPCR	Detection of WNV RNA [59]	Whole blood, serum, CSF, post-mortem CNS [57,59]	Highly specific; allows lineage identification; confirms acute infection [60]	Very short viraemia in horses reduces sensitivity; negative result does not exclude disease; requires specialised lab [57]
Conventional RT-qPCR	Detection of viral RNA [61,62]	CNS tissues post-mortem, blood (early) [61,62]	Useful for molecular epidemiology; lineage differentiation [61,62]	Lower sensitivity than real-time PCR; limited value in living horses outside very early infection [61,62]
Virus isolation	Replication of live virus in cell culture [63]	Brain, spinal cord, occasionally blood [62,63]	Definitive confirmation; enables full genomic analysis [62,63]	Requires BSL-3 facilities; slow; low sensitivity due to low viraemia; rarely used in routine diagnostics [62,63]
Immunohistochemistry (IHC)	Detection of viral antigen in tissues [64]	CNS tissues post-mortem [64]	Confirms infection in fatal cases; useful when RNA degraded [49,64]	Only applicable post-mortem; dependent on tissue quality [49,64]
IgM capture ELISA	Detection of IgM antibodies indicating recent infection [8]	Serum, CSF [8]	Best marker of acute/early infection; practical and rapid [8]	IgM short-lived; titres variable; potential cross-reactivity; may miss infections if sampling delayed [11,31]
IgG ELISA/cELISA	Detection of IgG antibodies indicating past exposure [31,65]	Serum [31]	Suitable for serosurveys; high throughput; widely available [31]	Cannot distinguish recent vs. past infection; cannot differentiate infection from vaccination; cross-reactivity [12,65]
Virus neutralisation test	Functional neutralisation of WNV by serum antibodies [66]	Serum [8]	Gold standard for specificity; resolves cross-reactivity [8,63]	Labour-intensive; requires virus manipulation; slow turnaround; not widely available [8]
Western blot (rarely used)	Detection of antibodies against specific viral proteins [67,68]	Serum [67,68]	High specificity in confirmatory use [67,68]	Not standardised for equines; limited availability [67,68]

## 4. Surveillance Systems in Equines

Surveillance of WNV in equine populations represents a key component of integrated monitoring frameworks aimed at detecting viral circulation, assessing regional risk and supporting early warning systems for both veterinary and public health authorities [69]. Because horses are highly susceptible to WNV and share ecological exposure with humans, data derived from equine surveillance can provide valuable indicators of local transmission intensity and the emergence of new hotspots [32].

Equine surveillance typically relies on three complementary approaches: passive clinical surveillance, active serological monitoring and sentinel-based surveillance.

Passive clinical surveillance remains the foundational strategy in most countries. It relies on the reporting of horses presenting with neurological signs compatible with WNV infection, followed by targeted diagnostic testing. This method is cost-effective and directly linked to clinically relevant disease; however, its sensitivity is limited by under-recognition, inconsistent reporting practices and the variable awareness of owners or veterinarians. As a result, many subclinical or mild cases remain undetected, leading to an underestimation of the true level of viral circulation [70,71].

In contrast, active serological surveillance aims to detect past exposure by screening healthy equine populations for the presence of IgG antibodies. Cross-sectional surveys can quantify seroprevalence across ecological zones, while repeated sampling enables the identification of temporal trends in exposure risk [11,12]. This approach is particularly useful for detecting silent circulation and identifying areas where viral amplification is occurring in the absence of clinical disease. Nevertheless, it requires structured sampling designs, adequate laboratory capacity and sustained financial resources, and results must be interpreted in the context of regional vaccination practices and potential cross-reactivity with other flaviviruses [30,65].

Sentinel equine surveillance, based on periodic sampling of unvaccinated cohorts, provides an enhanced capacity for early detection of viral introduction or resurgence. Seroconversion in sentinel horses often precedes the occurrence of clinical cases, making this approach a valuable early warning tool in high-risk areas such as wetlands, irrigated agricultural zones or regions with high mosquito density. However, successful implementation requires consistent sampling intervals, careful selection of sentinel groups and reliable access to diagnostic assays capable of distinguishing recent infection [32].

To maximise epidemiological insight, equine surveillance should be integrated within broader One Health systems that incorporate mosquito monitoring, avian surveillance and environmental data [72,73]. Linking equine seroconversion with entomological indices, climatic indicators and bird migration patterns generates a more comprehensive understanding of local transmission dynamics [74,75]. When combined with spatial modelling and risk forecasting, integrated datasets can support targeted vector control measures, guide veterinary alert systems and improve preparedness for human health authorities [76,77].

**Figure 3:**
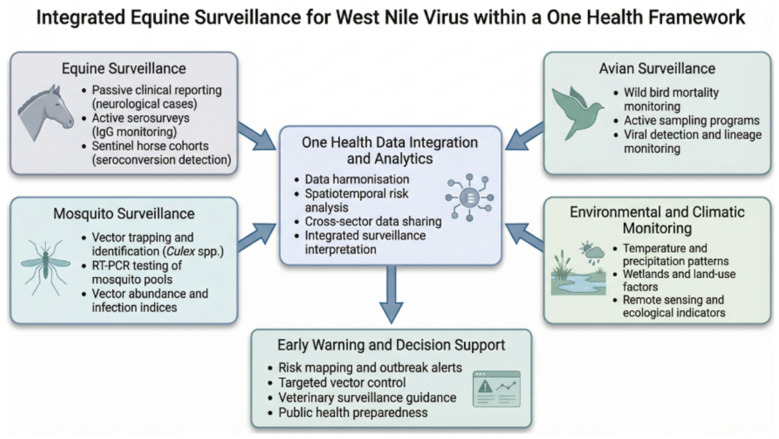
Integrated equine surveillance components within a One Health framework. This figure presents the major elements of equine WNV surveillance within a One Health system. Passive clinical surveillance detects neurological cases in horses, while active serological monitoring reveals silent transmission. Sentinel equine cohorts provide early warning of viral amplification. Integration of equine data with mosquito surveillance, avian monitoring and environmental indicators enhances risk assessment, supports predictive modelling and enables timely public health response.

Despite these advantages, equine surveillance across Europe remains heterogeneous [9]. Differences in assay selection, diagnostic interpretation, sampling frequency and reporting channels limit cross-national comparability and hinder the development of harmonised early warning frameworks. Strengthening laboratory coordination, establishing standardised protocols and improving data exchange between veterinary and public health sectors are essential steps for enhancing surveillance effectiveness and supporting accurate risk assessment [78]. The main surveillance approaches for WNV in equines, including their characteristics, strengths and limitations, are summarised in Table 2, while the main components of equine WNV surveillance within an integrated One Health framework are illustrated in Figure 3.

**Table 2:** Surveillance approaches for WNV in equines: characteristics, strengths and limitations.

Surveillance Type	Description	Goals	Strengths	Limitations
Passive clinical surveillance	Reporting and testing of horses with neurological signs [70]	Detect clinical cases; signal active circulation [11]	Low cost; directly linked to disease burden [79]	Misses subclinical infections; dependent on reporting [70]
Active serological surveillance	Cross-sectional or longitudinal IgG screening of healthy horses [7,31]	Assess exposure, identify silent transmission [7,31]	Detects asymptomatic infections; reveals hotspots [7,31]	Requires structured sampling; laboratory capacity; cost [7,31]
Sentinel equine surveillance	Monitoring unvaccinated horses for seroconversion [74,80]	Early detection of viral amplification [74]	Sensitive, predictive; valuable for early warning [74]	Requires regular sampling; logistical coordination [74,81]
Integrated One Health surveillance	Combining equine, mosquito, avian and environmental data [9]	Comprehensive risk assessment; outbreak prediction [9]	Most informative and robust; improves preparedness [14]	Requires cross-sector collaboration; variable data quality [82,83]
Post-mortem surveillance in fatal cases	Necropsy + IHC/PCR of CNS tissues [80]	Confirm fatal cases; detect severe outbreaks [84]	High diagnostic accuracy [49]	Only detects severe/fatal infection; small sample size [85]

## 5. Challenges in Diagnosis and Surveillance

Despite notable advances in understanding WNV epidemiology and equine infection dynamics, several biological, methodological and operational challenges continue to limit the accuracy of diagnostic testing and the effectiveness of surveillance systems [10]. These constraints vary across regions and directly influence the timely detection of viral circulation, the interpretation of serological and molecular data, and the comparability of results across laboratories [9,10].

A major diagnostic limitation stems from the intrinsic characteristics of WNV infection in horses. Equines typically develop a short and low-level viraemia that frequently falls below the analytical sensitivity of nucleic acid amplification tests [8]. As a result, RT-qPCR performed on blood or CSF often yields negative results even in clinically compatible cases, particularly when sampling occurs after the early phase of infection [86]. Consequently, molecular assays cannot be relied upon as standalone diagnostic tools in equine medicine and must be interpreted in conjunction with serology and clinical findings [86].

Serological testing, although essential, introduces additional interpretative complexities [8]. IgM capture ELISAs are considered the most informative for detecting recent infection [86]; however, the magnitude and duration of IgM responses vary among individuals, and titres may decline rapidly, leading to false-negative results when sampling is delayed [38,87]. IgG ELISAs provide valuable information for seroprevalence studies but cannot distinguish between recent and past infection, nor can they differentiate natural exposure from vaccine-induced immunity in regions where vaccination is implemented [88]. Cross-reactivity with other flaviviruses, especially Usutu virus, further complicates serological interpretation and underscores the need for confirmatory testing using virus neutralisation tests (VNTs), which remain resource-intensive and are not widely available in all veterinary laboratories [50,88,89].

Beyond methodological constraints, significant heterogeneity exists in laboratory capacity, assay selection, cut-off values and confirmatory protocols across countries [9]. This variability affects the comparability of results and limits the generation of harmonised epidemiological indicators at the national or regional scale [10]. Differences in sample handling procedures, test validation and the availability of reference laboratories also contribute to inconsistent diagnostic performance and reduced confidence in surveillance outputs [8].

Operational challenges further hinder surveillance efficiency [89]. Passive clinical surveillance relies on the recognition and reporting of neurological signs by owners and veterinarians, yet mild, atypical or early cases may go unnoticed or remain untested [11,90]. Active serological surveillance and sentinel horse programmes offer greater sensitivity for detecting silent circulation but require sustained funding, structured sampling strategies, logistical coordination and long-term institutional commitment [91]. In resource-limited regions, such programmes may be implemented intermittently or discontinued, resulting in spatial and temporal gaps in surveillance data [91].

Environmental variability adds additional complexity [20]. Rapid fluctuations in climatic conditions, vector abundance and avian reservoir dynamics can lead to abrupt changes in WNV transmission intensity [35]. Surveillance systems that lack sufficient temporal resolution, geographic coverage or cross-sector integration may fail to detect these shifts in real time [36]. Moreover, the absence of standardised thresholds for triggering public health or veterinary interventions complicates decision making and reduces the operational value of equine-derived indicators [92].

**Figure 4:**
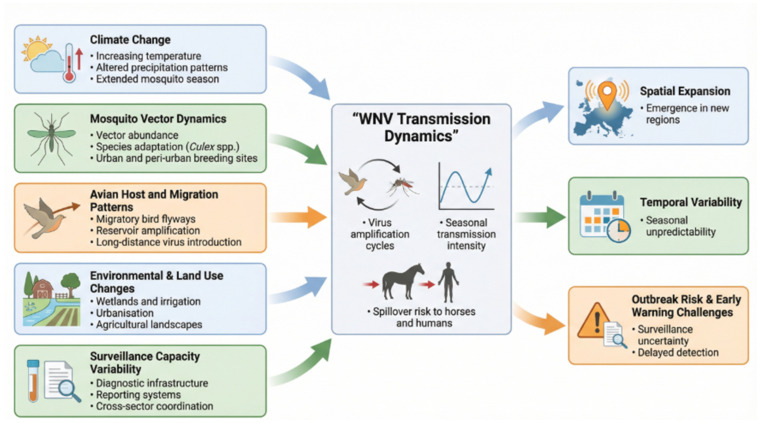
Key ecological, climatic and operational determinants shaping WNV epidemiology in Europe. This conceptual map summarises major determinants influencing WNV transmission dynamics in Europe, including climate change, mosquito vector adaptation, bird migration, urbanisation and variability in surveillance capacity. These interacting factors contribute to the spatial expansion, temporal variability and unpredictability of WNV outbreaks and must be considered when designing surveillance strategies and interpreting equine diagnostic data.

Overall, these challenges highlight the need for improved diagnostic standardisation, enhanced laboratory coordination, strengthened One Health data integration and sustained investment in targeted surveillance [93]. Addressing these limitations is essential for accurately characterising WNV transmission patterns, improving early warning capabilities and reducing the disease burden in equine populations [36]. The main diagnostic challenges and their implications for equine surveillance are summarised in Table 3, while the key ecological, climatic and operational determinants shaping WNV epidemiology are illustrated in Figure 4.

**Table 3:** Key diagnostic challenges and implications for surveillance in equines.

Challenge	Description	Impact on Diagnosis	Impact on Surveillance
Short and low-level viraemia	Typical for equine WNV infection [8,94]	Low sensitivity of PCR; frequent false negatives [8,94]	Underestimation of active viral circulation [8,94]
Serological cross-reactivity	Overlap with other flaviviruses (e.g., Usutu virus) [88]	Ambiguous IgG results; need for VNT confirmation [88]	Inflated seroprevalence estimates if unconfirmed [88]
Variable IgM kinetics	IgM appears early but may decline rapidly [95]	Missed acute infections if testing is late [95]	Reduced ability to detect recent cases [95]
Vaccination interference	Vaccinal antibodies resemble natural infection [87,96]	Difficult to distinguish true infection [87,96]	Complicates interpretation of serosurveys [87,96]
Laboratory heterogeneity	Differences in kits, cut-offs and capacity [9]	Inconsistent results across regions [9]	Inconsistent prevalence estimates [9]
Limited reporting	Low awareness or recognition of neurologic signs [91]	Cases untested or unreported [11]	Weak passive surveillance signals [11,91]

## 6. Future Directions

Future advancements in the diagnosis and surveillance of WNV infection in equines will rely on the integration of novel technologies, improved diagnostic standardisation and the strengthening of One Health-oriented monitoring frameworks. Several priority areas emerge from the current knowledge gaps [8,73].

The enhancement of diagnostic tools remains essential. Although molecular assays show high analytical specificity, their clinical sensitivity in equines is limited by the short and low-level viraemia characteristic of infection [37,97]. Future progress should focus on developing rapid, field-deployable molecular platforms, such as portable RT-qPCR units, CRISPR-based detection systems or loop-mediated isothermal amplification (LAMP), that maintain high sensitivity even in low-titre samples [98]. In parallel, serological tools require refinement to overcome issues of cross-reactivity with related flaviviruses and to better differentiate recent infection from past exposure or vaccine-induced antibodies [8,85]. Novel ELISA formats, improved antigen targets and well-characterised reference panels are needed to support assay harmonisation [8].

The standardisation of laboratory methodologies across regions will be equally important. Significant variability currently exists in ELISA cut-off values, confirmatory testing strategies and access to virus neutralisation tests (VNTs) [8]. Collaborative validation studies, harmonised protocols and shared quality assurance programmes would enhance diagnostic comparability and improve the interpretation of equine surveillance data at national and international levels [8].

From a surveillance perspective, the expansion of integrated, multi-source monitoring systems represents a major opportunity. Facility-level environmental risk scoring can provide a rapid, low-cost proxy for mosquito-favourable conditions and may complement entomological and serological surveillance to improve prioritisation and early warning capacity, particularly in resource-limited settings [99]. Linking equine serological data with mosquito surveillance, avian testing, climatic indicators and ecological modelling can substantially improve early warning capacity [73]. Advances in remote sensing, machine learning and climate-based prediction models could support the identification of high-risk areas, optimise sampling strategies and guide timely vector control interventions [76]. In this framework, equine sentinel cohorts, particularly in ecologically sensitive or high-transmission regions, can provide sensitive and cost-effective indicators of viral amplification when coupled with regular sampling and robust diagnostic support [74,80].

Additionally, genomic surveillance is expected to play an increasingly prominent role. High-throughput sequencing of WNV-positive samples, even at low coverage, can elucidate viral lineage dynamics, track geographic spread and detect emerging mutations associated with virulence, host adaptation or immune evasion [100]. Incorporating equine-derived genomic data into broader European or international phylogenetic networks would enhance situational awareness and support coordinated public health and veterinary responses [101].

Finally, long-term improvement requires structured investment in training, infrastructure and intersectoral collaboration [102]. Strengthening communication between veterinary laboratories, public health agencies, wildlife surveillance programmes and environmental monitoring systems is essential for operationalising One Health strategies [103]. Improved funding mechanisms, cross-border data sharing and coordinated policy frameworks will further support sustained and effective surveillance.

Collectively, these future directions highlight the need for innovative diagnostic methods, harmonised laboratory practices and integrated surveillance approaches tailored to evolving ecological and epidemiological contexts. Implementing these priorities will be critical for enhancing preparedness, reducing disease impact in equine populations and refining the early detection of WNV circulation at the human–animal–environment interface.

## 7. Conclusions

WNV infection remains a significant and evolving threat to equine health, with implications extending beyond veterinary medicine into the wider One Health domain. Horses, although incidental hosts, develop severe neurological disease more frequently than humans and therefore provide an early and sensitive indication of viral circulation in the environment. Their clinical and serological responses offer a practical means to detect changes in transmission intensity, making equine surveillance an essential component of integrated monitoring systems.

Despite notable advances in understanding WNV epidemiology in Europe, major gaps persist in both diagnostic practice and surveillance implementation. The short and low-level viraemia characteristic of equine infection limits the usefulness of molecular assays in clinical cases, necessitating a continued reliance on serological tests. However, cross-reactivity with other flaviviruses, variability in IgM kinetics and difficulties in distinguishing natural infection from vaccine-derived immunity complicate serological interpretation. These constraints highlight the need for standardised diagnostic algorithms that combine clinical presentation with carefully selected laboratory methods.

Surveillance systems have expanded in recent years, but substantial heterogeneity remains across Europe. Differences in laboratory capacity, sampling frequency, case definitions and reporting structures impede comparability and weaken early warning capabilities. Integrating equine data with mosquito surveillance, avian monitoring and environmental indicators represents a critical step toward more accurate risk assessment and predictive capability. Harmonised procedures and improved data-sharing mechanisms are essential for building an efficient, coordinated surveillance infrastructure.

Looking forward, investment in diagnostic innovation, including rapid point-of-care tools, improved serological assays and lineage-specific molecular tests, will be important for strengthening field-level detection. Likewise, adaptive surveillance frameworks that incorporate modelling, remote sensing and climate-based forecasting will be needed to address the increasingly dynamic nature of WNV transmission under changing ecological conditions.

Overall, enhancing diagnostic performance, standardising surveillance protocols and embedding equine data more systematically into One Health systems will be crucial for improving outbreak detection, response efficiency and preparedness for future WNV transmission seasons.

## Data Availability

No new data were created or analysed in this study.

## Abbreviations

The following abbreviations are used in this manuscript:

WNV	West Nile virus
CSF	Cerebrospinal fluid
CNS	Central nervous system
ELISA	Enzyme-linked immunosorbent assay
IgM	Immunoglobulin M
IgG	Immunoglobulin G
PRNT	Plaque reduction neutralisation test
BSL	Biosafety level
ECDC	European Centre for Disease Prevention and Control
EFSA	European Food Safety Authority
